# Supplementary material for: Targeting Sex Determination to Suppress Mosquito Populations
Source: Res Sq. 2023 Apr 24:rs.3.rs-2834069. Preprint. [Version 1] doi: 10.21203/rs.3.rs-2834069/v1 (PMC10168471; doi:10.21203/rs.3.rs-2834069/v1)
Supplement: Supplement 1 [file NIHPPRS2834069V1-supplement-1.pdf]

Supplementary Files

This is a list of supplementary files associated with this preprint. Click to download.

- SUPPLEMENTALDATA.docx
- Sourcedata.xlsx
- SupplementaryFigures.docx
